# The Dietetics of the Soul
*"The Dietetics of the Soul." By Ernest Von Feuchtersleben, M.D. Edited from the Seventh Edition, pp. 202. London: J. Churchill.


**Published:** 1853-07-01

**Authors:** 


### Art. II.—THE DIETETICS OF THE SOUL.*

In one of those beautiful phrases which have won for him the title of 'divine,' Plato expostulates thus with his soul, under the appellation of a Star,

> Αστέρας εἰσαθρεῖς, ἀστηρ ἐμός· εἴθε γενοίμην οὐρανὸς,
> ὡς πολλοῖς ὀμμασιν εἴς σε βλεπω.

This Star, or rather ψυχὴ remains as great a mystery now, as then. The broad-browed philosopher was unable to gratify the yearnings of his mighty soul by a knowledge of its own intrinsic nature, and the lapse of two thousand years has found his followers beset by the same aspirations, and involved in the same ignorance. The ἀστηρ ἐμός—the 'Ego,' is unsolved and unsolvable. The patient observation of centuries, and the untiring speculations of philosophy, have thrown no light upon this mysterious essence. The definitions of Plato are little

* "The Dietetics of the Soul." By Ernest Von Feuchtersleben, M.D. Edited from the Seventh Edition, pp. 202. London: J. Churchill.

less obscure than the subtleties of Fichte and Hegel, and almost as elucidatory as the coarser theory of Cabanis and Gall. Has the science of Psychology remained stationary in this age of progress and change? By no means. Its followers have long ago learned to study facts rather than "*essences*," and to be content with a knowledge of phenomena, rather than to grope darkly on for ever, in the vain hope of discovering the precise character of the "*noumenon*," or "substance," the "*Esse*," the primal self, upon which the philosophers of old were so fond of weaving mystic, cloudy, and useless speculations. And yet, these great thinkers were not devoid of observing power. Scintillations of great truths may be found, here and there, in the writings of most of them, and their exertions have smoothed the path of modern discovery. They have been to Psychology what the Chaldeans and Astrologers were to astronomy, or the alchemists to chemistry. They have taught us what to avoid, and have chronicled many facts of great importance. They have been the pioneers of Truth, and therefore we would cherish their names with reverence, as we would the memory of a friend. Among the facts observed and registered is the compound character of mental manifestations, and the subtle connexion of the soul with the body; thus Aristotle states of the soul, that "it is not the body, but somewhat of the body," and yet in other writings regards it as distinct and separable—(*ουσια χωριστη και κεχωρισμενη των αισθητων*)—recognising, however, two distinct manifestations of intelligence, the one as active (*νους*), the other passive—that imperishable, this the subject of death (*τουτο μονον αθανατον και αϊδιον, ο δε παθητικος φθαρτος.*) Still earlier, indeed, in the history of metaphysics, we are able to trace this idea of two or three faculties presiding over, or rather constituting mind, for even Pythagoras enumerates three as distinct, thus—reason (*νους*), intelligence (*φρὴν*), and desire (*θυμὸς*); and this idea becomes more and more distinct as we reach the Christian era. In the Inspired Writings, man is constantly referred to as a dual (or rather a triple) entity when *the whole man* is spoken of; if Luke, the beloved *Physician,* be writing, we still read of "soul" and "spirit" (*ψυχὴ καὶ πνεῦμα*) in his record of the song of the Blessed Virgin, as we do when Paul the tent-maker breathes a fervent prayer that the Church of the Thessalonians may be preserved blameless both in spirit, and soul, and body (*τὸ πνεῦμα καὶ ἡ ψυχὴ καὶ τὸ σῶμα*) or when the unknown writer of the Epistle to the Hebrews gives us a thrilling account of the power of God's word in the dividing asunder of "soul and spirit" (*ψυχῆς τε καὶ πνεύματος*). The modern phase of Psychology, as represented by Fichte, is not so conformable to each man's experience as the above, and we regard the "Absolute Idealism" of Hegel, with a profound writer of the present day, as nothing more than "Hume's scepticism in a dogmatic form."

Hegel's hypothesis may be regarded as an error on the opposite pole to the Phrenological theory. Hegel is defective by disregarding, or rather denying "matter," maintaining that "the things we know are not only appearances to us, but are *in themselves* mere appearances (sondern ansich blosse Erscheinungen)," thus making the mind everything—"that our thoughts are not only thoughts, but at the same time are the reality of things." Hegel has many able disciples, whom these remarks may possibly offend; but we must be loyal to truth. Phrenology, on the other hand, we regard as mainly defective through overlooking the internal consciousness—the spiritual entity, the το πνευμα of the inspired writers. The followers of Gall practically ignore "The Will," that great faculty which has enabled the small head to succeed where the large head has succumbed to despair, or indifference—the faculty which all earnest souls have endeavoured to bring into obedience to certain duties, which the intellect has recognised as right, and whose struggles have been eloquently portrayed in the lives and writings of such illustrious men as Ignatius Loyola, Luther, and Bunyan, and, to seek a still higher illustration, is described with such truthful energy in the Epistles of St. Paul. There is nothing in the system to explain the "*my*" imagination, *my* consciousness, and the like; and regarded somatically it errs in leaving a large portion of the cerebral organ unappropriated, although all known psychical faculties are professedly embraced, and have been ingeniously arranged and allocated. The present generation, however, owes much to the genius of Gall and the zeal of his disciples, Spurzheim and Combe: for, to say nothing of their great anatomical discoveries, there has sprung up from their teachings much valuable knowledge on the important subjects of education and crime, and the close relationship between physical conformation and the manifestations of the *understanding*. Perhaps we do not exceed the limits of sober criticism, if we state that this theory contains the nucleus of a great truth, and that its fundamental principles have been demonstrated. It was the misfortune of Gall to have his theory become popular. The itinerant lecturers who followed in his wake, with a mental philosophy made easy for the million, have brought the theory into disrepute; and their rash and empirical practice of estimating the character of every individual who approached them, from the external configuration of his or her cranium, disgusted the thoughtful and the religious, and have done more than anything besides to obscure (or rather to bury) the vast array of facts upon which the *principle* of the theory is based. Its votaries had not been trained in the cautious school of philosophic induction. They endeavoured to build up a magnificent edifice in a short space of time, and in their haste were so regardless of the materials they used, or of the means of procuring cohesiveness, or adhesion of the parts, that the superstructure has tottered and fallen, and, for the present, have obscured the solid foundations of a great truth—a great truth, which, like all other great truths, had been dimly descried by many minds, before it was perceived and demonstrated by Gall. As left by Gall and Spurzheim the theory was necessarily imperfect, as imperfect as the theory of gravitation when it first dawned upon the mind of Newton, after the conjectures of Bullialdus, Hooke, and Halley, but, like that theory, we hesitate not to say, that its *principle* will hereafter receive confirmation and universal acceptance; the principle, as we understand it, being, that the brain is the organ through which mind displays itself—that this organ is compound in its character, and that different mental faculties act through different portions of this compound structure, and, *cœteris paribus,* the larger the organ the greater will be the intellectual manifestations of the individual *to whom* it belongs. As we have before intimated, the cardinal error in the system is the neglect of this "ego;" the system relates exclusively to the "*understanding,*" and reaches not the depths of humanity. Many of its details are fabulous. The faculties of the mind, as described by Gall and Spurzheim, are *popularly* understood as a *new* arrangement discovered by Gall, whereas Gall's great merit consists in his endeavours to allocate each faculty in separate parts of the brain. Other men had entertained somewhat similar notions. In a "Chart of the Universe and the Elements of all the Sciences" at the British Museum, bearing date 1632, there is a head mapped out into divisions; and there is now before us a head copied from a Venetian drawing, bearing date 1562, in which "common sense" is placed in the frontal region, fancy and imagination behind this; the reflective power still more posteriorly, and "memory" is located in the cerebellum. Gall elevated these crude notions into a "system," by his great work "Sur les Fonctions du Cerveau," and thus became the propounder of a theory which contains the nucleus of important truths. From this era we may date the commencement of the *popular* study of psychology. It is the start-point of that literature which, as we stated in our last number, "promises to become fashionable." These popular products vary greatly as to the ability and morality displayed in them,—from the bold and startling, yet clever little volume on "The Constitution of Man," by George Combe; the romances of electro-biology, and that curious compound of childish credulity and rash scepticism, "The Laws of Man's Nature and Development" (the rickety offspring of Harriet Martineau and William George Atkinson), up to the little work, whose peculiar phraseology and quaint title, "The Dietetics of the Soul," have elicited from us the above preliminary remarks. It is a translation of a *sixth* German edition,—the author being Ernest von

Feuchstersleben, the distinguished Psychological writer; the Translator is (for the present) anonymous, although it may not be difficult to guess his name. The work is avowedly popular in style, although its aims are high, and addressed to all classes. It is divided into thirteen chapters, as follows:—"The Introduction. 2nd. The Idea—General Actions of the Mind. 3rd. Beauty—The Reflexion of Health. 4th. Imagination. 5th. Will—Character—Indecision—Ill-humour—Distraction. 6th. Understanding—Culture. 7th. Temperaments—Passions. 8th. The Affections. 9th. Oscillation. 10th. Hypochondriasis. 11th. Nature—Truth. 12th. Resume. 13th. Passages from a Diary." The high respect in which the author is held throughout Europe, has prompted us to read the little work with great attention, and to bring its claims before our readers.

The Introduction is brief and desultory. It commences in a dirge-like strain, on "the stormy and unsettled times in which we live"—and states that a benefit may be conferred "not only on ourselves, but on others, by diverting attention from the exciting circumstances of the present day—from the disheartening eccentricities of a literature which meanders in a thousand frivolous directions—to the calm regions where the inner-man, self-examined, submits himself to moral treatment," and, it adds, after mourning over "the noise and dust, and highway reality" in the works of present authors, "which has been attained at the expense of that profundity of thought and *clearness of expression* which distinguished the writings of our forefathers," that "the following pages have been written to correct the tendencies just alluded to. They have been conceived in the spirit of repose, for self-cure, and self-meditation; and it is thus they must be read, if the reader would derive any benefit from them."—page 3.

The author deals very dogmatically, with both the terms "dietetics" and "soul." "The fact of consciousness, which is only recognised by the aid of inward reflection, or self-analysis, points to a principle different from those derived through perception. This principle we denominate *mind;* but it must not be forgotten that the word "mind" merely represents an abstraction; for in this world mind only appears to us through its manifestations in man—that is to say, in corporeal beings. When *thus associated with matter,* we term it in ordinary language *the* soul; and the substance united with the soul we denominate the body.—p. 12. This is a different distinction to that made in "ordinary language" by the people of this country—"the abstraction" represented "by the word, mind"—would here be called "*soul,*" and the term "mind," applied to it, so long as "it was associated with matter." The writer further remarks that "under the term, Dietetics of the Soul," I would comprehend a knowledge of those means *by*

*which the soul is preserved in a state of health.* This knowledge constitutes morality; and although all the mental efforts of man tend towards the same great aim of cultivating and fostering his mental sense, * * * yet I would here especially consider that power of the *mind by which it is enabled to avert the ills that threaten the body.* . . . This is the "Dietetics of the Soul," of which I propose to treat. Kant examined "the power of the mind to master morbid feelings by the mere force of resolution." I would go still further than this; and show not only how the feelings, but the excess of disease itself, may be controlled. The body is frequently the only channel through which we can assist the mind; but why may we not sometimes influence the former, through the latter? It may be, that neither medical men, nor the public—and here each man should be his own physician—have bestowed on this matter the attention which it merits. My object in the present work is to explain how this spiritual portion of man may be protected from disease."—p. 7—9.

We do not forget, that it is a *translation* that is before us, but it is the translation, as published in England, that we have especially to analyze. The hurry of the age has been condemned in the opening pages, and the absence "*of clearness of expression*" in modern writers been mourned over as its consequence. The *present* work "was written to correct such tendencies," and "was conceived in a spirit of repose." The object of the work, whether it be to teach us "the means by which *the soul* is preserved in a state of health," or "to consider that power of the *mind* by which it is enabled to avert the ills that *threaten the body*" (whether this or that really constitutes the "Dietetics of the Soul") is one of too great importance to be set aside, in consequence of the ambiguity of the style in which it is announced; but we were fairly entitled to greater "clearness of expression" in a work, whose opening pages are occupied in condemning the obscurities of other writers, and which arrogates to itself the task of correcting such tendencies. We will, however, pass over these defects for the present, and endeavour to reach the meaning of the writer, from the passages which follow.

"The happiness or misery of the individual depends on the deeply marked impressions or conceptions of his own mind. It is impossible to subject these impressions to control, or to obtain clearness of mental vision? We employ efforts enough to render it obscure. The wild fury of the storm which drenched Lear's companions to the skin, touched not the unhappy man himself, because an internal tempest of passion deadened the senses to all external impressions.

"Yet the most convincing proof of the strength of mind is, strange to say, to be found in its impotency. Every one knows that the unfortunate persons whose minds are buried in the night of insanity, remain exempt from many diseases which attack others around them; their minds are concentrated on some delusion—their attention diverted from bodily suffering; and *thus they are rendered insensible to external influences.* 'And shall not a cultivated, well-directed volition, have as much, nay greater, power than furious anger, or the horrible energy of the insane.'"—pages 16, 17.

We entirely demur to the premises above stated, in reference to these "unfortunate persons whose minds are buried in the nights of insanity." Nay more, it is a popular fallacy at variance with well-observed facts. When cholera is ravaging a district, are the insane exempt—"are they rendered *insensible* to external influences?" In the early cholera reports from India, we are informed, that they were among the first of its victims. In 1849, cholera was introduced into the Wakefield Asylum, Yorkshire, by a patient brought from a workhouse in which the disease existed,"—and the result was, that out of 620 patients then in the Institution, 200 became attacked with diarrhœa—27 with dysentery, and 132 by cholera. This epidemic raged *virulently* for ten days, and in one day no less than 19 patients died; it extended over six weeks, and destroyed 98 patients. No form of mental disease "exempted" the patient "from this external influence," as the following table by Dr. Wright abundantly testifies:

| | | | | | | |
|---|---|---|---|---|---|---|
| Delusions . . | 34·2 per cent. | were ill. | 14·2 | per cent. | had cholera. |
| Mania . . . | 47·8 | " | " | 22·7 | " | " |
| Melancholia . | 55·5 | " | " | 11·1 | " | " |
| Dementia . . | 55·4 | " | " | 22·7 | " | " |
| Amentia . . | 42·0 | " | " | 20·0 | " | " |

The supposed exemption of the insane from typhus fever may have arisen from their being isolated from its influence; and from some other diseases, by the counteracting effect, which pre-existing *corporeal* ailments sometimes exert over the approach of another malady, rather than from any direct psychical cause. We are certain that exposure to cold, to damp, and other mischievous agents, produces pulmonary affections, as in the sane, although these affections may not be characterized by the same vital symptoms, such as coughs, and the like. We entirely demur to the strong antithetical sentence, that "the most convincing proof of the *strength* of the mind is to be found in its *impotency*," for in those cases of marvellous anæsthesia, with which the annals of psychology abound, some individual passion is beset with morbid force, and exerts an energy comparable only to some forms of muscular action under tetanic spasm, and in this condition will exert a far greater power than any cultivated, well-directed volition whatever. In stating this, we have not forgotten the heroism of Regulus—the sublime courage of "The Holy Army of Martyrs,"—or the noble pride which wiped away—

. . . "The last—the first—
The only tears that ever burst
From Outalissi's soul;"

Indeed, the reflecting reader will observe, that in all these cases, there is one powerful mental emotion placed in antagonism with another, and that the physical suffering is defied and concealed, rather than unfelt. When, under the influence of a noble patriotism, Regulus mocked the torments of the Carthaginians,—or Cranmer thrust his arm into the burning flame at Smithfield, the sufferings consequent upon these procedures were not extinguished by these acts of powerful volition, but simply concealed. There is also a radical difference between sufferings inflicted from without *by others,* and the sufferings which spring up from the operations of disease within the body of the sufferer. By the one, the mental energy is roused; by the other it is often depressed, and that, too, in defiance of the strongest volitional power to the contrary. The storm beating around Lear, *was felt* by the passion-tossed man, but being in harmony with his own tumultuous feelings, he could bid the winds to blow,—"and crack your cheeks! rage! blow!"—and yet, nevertheless, his senses were not *dead* to their presence, as exhibited by the following expressions full of feeling, and apparently of just upbraiding:—

> "I tax not you, you elements, with unkindness,
> I never gave you kingdoms, call'd you children,
> You owe me no subscription; why then, let fall
> Your horrible pleasure; here I stand your slave,
> A poor, infirm, weak, and despised old man:—
> *But yet I call you servile ministers*
> *That have with two pernicious daughters join'd*
> *Your high-engendered battles 'gainst a head*
> *So old and white as this. O! O! 'tis foul.*"

Again:—

> "How dost my boy? Art cold?
> *I am cold myself.*"

And even after the celebrated speech, which is in itself so truthful, but which has elicited so much false inference, he tells us—

> "This tempest *will not give me leave to ponder*

> *On things would hurt me more.*"

Hence, its acceptability and welcome. We do not object, however, to the principle enunciated, viz: the great power which a strong volition is capable of exerting over many diseases of the body, and some affections of the mind; but we regard the passages above quoted, as conveying *exaggerated,* and therefore false notions of this power. A love of paradox has also tended to obscure the meaning of the author, as we have shown by quoting his varying statements, respecting the "Dietetics of the Soul." The great poet of all time has enunciated the philosophy of this little work in the following lines:

"'Tis in ourselves that we are this or that, our bodies are our gardens, to which our wills are gardeners, so that if we will plant nettles, or sow lettuce, or set hyssop, and weed up thyme, either to have it sterile with idleness, or manured with industry, why the power and corrigible authority of this lies in our wills. If the balance of our lives had not one scale of reason, to balance another of sensuality, the blood and baseness of our nature would conduct us to most preposterous conclusions."

Of late years, in England, whatever may have been the case in Germany,—this principle has been fully recognised and acted upon. Even many popular works have been issued to enforce it, and to show the power which the "soul has over the body;" and we have much pleasure in reminding the public of a most valuable little volume ofthi s kind, written by the learned Secretary of the Royal Institution, and entitled "Man's power over himself, to prevent or to control Insanity." It should not, however, be forgotten, that the body also has power over the Mind, and that there are several maladies, such as hepatic arrangements, and some affections of the heart—which are invariably characterized by symptoms of mental depression and timidity—and which feelings can only be made to disappear by remedies having direct reference to the physical disturbance. The records of every lunatic hospital will testify to the close association between bodily diseases and mental manifestations; for example, some forms of indigestion and melancholia, and the sympathy between disorders of the uterine function in young women, and acute mania; but the necessity of considering the reciprocal action of mind upon body, and body upon mind, is especially enforced upon the psychological physician by such facts as these:—

"A time arrived when the physician appointed to the Hospital *had no faith in medicine* in the treatment of insanity, *but relied chiefly upon moral treatment*, upon good diet and exercise, and upon the occasional use of purgatives for effecting a cure; and we find by referring to our tables, that the average per centage of recoveries during this period, *i. e.*, from 1791 to 1800, was 11½ *per cent. lower* than between 1831 and 1840. This fact alone, without reference to any other considerations, would have been sufficient to have convinced us of the importance of attending to the medical treatment of the patients confided to our care; and we are of opinion that the moral treatment being the same, and other things being assumed equal, the number of recoveries will advance *pari-passu* with the improvements in our knowledge of the pathology and medical treatment of the disease."—*Report of St. Luke's Hospital*, 1850.

A careful comparison between the cures in those asylums where the resident physicians avow their disbelief in the efficacy of medicine, and those hospitals in which all the resources of medical science are brought to the aid of the moral management, will show analogous results to those described in the Physician's Reports above referred to. We have no wish to undervalue "Moral Treatment." Our whole life has been devoted to its enforcement. But we fear from certain appearances, that a feeling has been fostered in the public mind by some good and worthy men, that any benevolent person is fitted to take the guidance of a lunatic hospital, however ignorant he may be of the great resources of medical science. Indeed this opinion was once practically carried out in the largest lunatic hospital of this kingdom. We believe the experiment was a failure, for its entire history, its beginning and its end, extended over but a few months. In the reports of the committee of that Hospital (which are not remarkable for their brevity), we read of the appointment of a *non-medical* gentleman to the office of Governor, who "*came recommended to them by a long and useful career of military service*"—and in about six months afterwards, we read again, that "the *Governor of the Asylum* tendered his resignation, which was accepted by the Visiting Justices." We revert to this singular incident in illustration of the principle, that a false doctrine is always followed by practical evils.

Feuchtersleben quotes and adopts the following statement:—"It is indeed well known that the dark days of November are the season of melancholy and suicide; but the gloom of the atmosphere cannot overcast the brightness of an unclouded spirit."—p. 18. We have elsewhere* shown, that this is rather a poetic fancy, than an established fact—the maximum of suicides, as far as statistical data can be ascertained, appears to occur in the months of June and July, rather than in November; and over a series of seven years, we found these sad catastrophes to happen about as follows—

| | |
|---|---|
| "In Spring | 997 |
| In Summer | 933 |
| In Autumn | 627 |
| In Winter | 648."† |

The "brightness of an unclouded spirit" can, alas! in sensitive natures, be "overcast" by certain atmospheric conditions; and happily, they can be sustained and elevated by other conditions of the same element. The great poet, who portrayed the beauties of the primeval Eden, and gave to us some glimpses of that brighter region, where sin is never triumphant, has assured us that his imagination was most active from September to the vernal equinox, and the lives of other poets furnish instances of a similar sensitiveness to external conditions. We

* "Anatomy of Suicide." London, 1840. † Ib. p. 132.

know that Dr. Johnson could deride this feeling as the fumes of a vain imagination, and yet in the pages of his 48th Rambler is found an opinion very like to it—"There are perhaps few conditions more to be pitied than that of an active mind labouring under the weight of a distempered body. The time of such a man is always spent in forming schemes, which *a change of wind* hinders him from executing . . . . . He lies down delighted with the thoughts of to-morrow, pleases his ambition with the fame he shall acquire, or his benevolence with the good he shall confer; but in the night *the skies are overcast, the temperature of the air is changed,* he wakes in languor, impatience, and distraction, and has no longer any wish but for ease, nor any attention but to misery." Contrasted with this, are the exhilaration and buoyancy felt by healthy persons on a fine frosty morning. Again, who has ever looked forth on a bright morning in June, when from every hedgerow on the earth, and even from the high vault of Heaven itself, the uprising sun was being greeted with melody and song; when myriad dewdrops were flashing back its splendours with all the radiant hues of the rainbow, and not felt a joy, a thrill, a transport, in marked contrast with the feelings which he has experienced, when the same sun has been sinking below the horizon, and the golden fringes of the purple clouds were being drawn around it, to the musical requiem of the solitary thrush? Genius is especially sensitive "to external influences." We would fain tell of how Canova missed his Italian sky, of the inspiration which Byron and Burns gathered from the tranquil landscape, the roaring tempest, or the sunny lake;—of clear "placid Leman," and the thunder-storm between Kenmuir and Gatehouse, which awoke the Tyrtean song of "Scot's wha hae;"—and under what "external influence" the great thought was engendered in the mind of Gibbon, to write "The Decline and Fall of the Roman Empire." We are tempted to record the birth-hour of many a deathless poem, of many a breathing marble, and of great historic deeds, but our space prevents the introduction of so fascinating a theme.

The chapter entitled "Beauty the reflection of Health," contains many paragraphs that are fanciful and false, blended, we are bound to state, with still more that are fraught with practical wisdom and sound morality. Among the former, we include the following hypothesis:—

"Here I found *my own opinions* expressed much more boldly than I should have ventured to state them. But why not quote the author's words? 'At present we can only venture to advance hypothetically, that a good man makes the air and earth around him healthy; while a bad man and a bad deed infest the scene of their action, causing the *virtuous to shudder,* and *the weak to incline towards evil, when they approach the spot.* Such ideas may seem quaint and superstitious at the present day; but after the lapse of another century, they may be regarded as truisms. Every one knows the popular belief respecting the spot on which a murder has been committed. Now, popular belief furnishes a rich and important source of knowledge respecting natural phenomena, because it results from the united experience of many, not from the reflections of a few. It is to be regretted, that we do not know whether Dr. Haine of Berlin, whose diagnostic powers rendered him so celebrated, and who could distinguish the various eruptions of the skin by their odour alone, *may not have been able to discover moral peculiarities by the same faculty.*"—page 25.

No German mind seems capable of freeing itself wholly from the mystic and transcendental; and such episodes as the above will more or less creep forth from the writings of the countrymen of Heinroth and Richenbach. The value of this little work must not, however, be estimated by such a paragraph as the above, for although it contains a few other sentences of a similar character, and is moreover radically defective by omitting to define the especial and essential characteristics of "the soul;" yet its aim is noble,—it contains many profound and suggestive ideas, and practical hints of great value. The following we regard as the utterances of a practical, masculine, and virtuous mind:

"We may seek in vain to enlighten the understandings of the insane, or convince them of the absurdity of their fixed ideas; but we may succeed in effecting a cure if we can excite into activity the faculty to will and to do."—page 54.

"No one can avoid being sometimes sad; but every one can eschew ill-humour. The former has a certain poetic charm about it; the latter is utterly devoid of attraction. It is the very prose of life, the sister of ennui and laziness. If we seek to trace the source of this poison from the experience of every-day life, we shall find that it depends on habit, 'that nurse of man' and of his vices. Accustomed from childhood to spend every superfluous hour in cheerful occupation, until the sweet, yet urgent demands of sleep compelled us to sound and healthy dreams, we should never have been ill-humoured. Were we never to waste the sweet morning hours in sleep, we should know nothing of that morose indolence which generally arises from the feeling of having slept too long. Did we habitually and constantly arrange everything around us with regard to cheerfulness and order, the same regularity would be harmoniously reflected in our souls. In a cheerful orderly apartment a man's feelings become cheerful; they partake of that which surrounds him. *But the best way* to avoid ill-humour is to *employ our leisure* moments in a proper manner."—page 62.

While, however, we admire the *morality* of the above, we have again to complain of the looseness of expression. In a work which "has been conceived in the spirit of repose," and has for one of its objects the correction of such "tendencies" in others; and commences its task, indeed, with a loud complaint against modern writers for this very fault, such errors call aloud for reproof. The German edition is not within our reach, so that we know not whether this besetting sin belong to Feuchtersleben or his translator; but its extent is great and obvious, as will be seen by our continuing the quotation :—

"*Religion*—that true knowledge of the love which should guide and accompany us at every step—will preserve its followers *from ill-humour more certainly than any other influence.* The disposition which receives all blessings with gratitude, will support evil fortune more lightly. When a man has had the misfortune to be born ill-humoured, he should not, as most do, deceive himself; he should rather regard himself as labouring under disease, and employ every means to get rid of the affliction."—page 62.

The remedy for this, and all other evils to which the author alludes, is "the force of volition." To those who object to the remedy as one beyond their reach, he replies :—

"If any one should object to this, that he is devoid of the force necessary to direct himself, I would recommend him to place himself in some position where he *must* act. This, at least, he can do. The first step is everything. A man may be without an occupation, and have no desire to enter on one. Let him devote himself to the state, or to some individual, in such a manner that he shall be compelled to work. By laying hold of the first *best* that offers, and cutting off all choice, we put an end to all vacillation; the melancholy cloud of torturing thoughts is at once dissipated by active, if unwilling, social employments; useless cares are thus thrown aside, and an apparent cheerfulness assumed, which eventually ends in becoming a real one. For the cure of mental diseases, writes a deep thinker, 'the understanding can do nothing, reason but little, time much, resignation and activity everything.' This mode of preventive or rather curative treatment, is based on the law that a strong stimulus must always displace a weaker one. When the mind, and through it the body, are acted upon by *the will*, the most diffusible and potent of all stimulants, other agents must be blunted and rendered comparatively innoxious."—page 59.

"So certain is it that the wondrous organization of man conceals unrevealed powers which an iron will may awaken and develop to astonishing perfection . . . . . . . . . . . . Cicero relates how a stoic philosopher, attempting to establish in the presence of Pompey that 'pain is no evil,' subdued a violent attack of gout in his own person, and demonstrated his argument, as it were, on his own feet. Was this act one of simple demonstration? Was it not rather the living sentiment of its import which effected the miracle? The Stoic school first taught its followers, by great examples, to exercise their volition: the latter having convinced themselves of the reality of this power, reflected on it, and handed down to us the simple but grand formula of doctrine —'What the spirit *wills*, the body *must*.'"—p. 66.

"To the victim of hypochondriasis I have but this advice to give. Turn your clouded sight from the narrow sphere of your own miserable tortured self, to the boundless theatre of suffering or rejoicing humanity; forget your own miseries in sympathy with your fellow-men; or at least deserve the sympathy of others. These are holy duties, which the great movements of the present day render incumbent on us all; and they are more easy of fulfilment than the *blasé* egoist, or the slave of habits can conceive. As a highly gifted poet and physician observes, 'Do we not feel ourselves when we feel for others?'—In the glory of ever-renovating and ever-living nature, the unhappy will find the consolation vouchsafed and prepared for all human beings; and in the conflicting maze of characters and destinies will he discover the place he was destined to fill. After this discovery nothing remains for him but to be and remain what his being prompts him to be—pure and truthful as the incorruptible word of God. Health is nothing but beauty, morality, and truth."—p. 157.

We have not given these lengthened quotations because we deem them novel, but because we conceive that they contain much truth,—The remedial effect of the "will," and the curative energy of "faith," have been long known and acted upon by English physicians. Our literature abounds with illustrations, and scarcely a number of this journal emanates from the press, without adding to them; as witness the elegant paper in our last, on "Psychotherapeia." We believe that the lists of mesmeric cures, of which we formerly heard so much, are traceable to the action of the imagination upon the body—and that Homœopathy is successful through the same medium. Viewed psychologically, this theory of Feuchtersleben's is faulty, inasmuch as he has wholly overlooked the power which many corporeal ailments have upon the energies of the will, and has nowhere drawn a proper distinction between the faculties of the understanding and the instinctive emotions which belong to the higher order of animals, and those endowments of the reason which are peculiar to, and the especial characteristics of the children of men.

When we commenced this analysis of "The Dietetics of the Soul," we purposed to illustrate the above defect at considerable length—but the many suggestive passages contained in the work, have so encroached upon our space, that we cannot, in justice to other works now before us, do more than make this passing allusion to them. It is always more delightful to us to discover beauties than to detect faults; to draw the attention of our readers to the great thoughts and noble aspirations of genius, rather than to its foibles or its follies, but the stern requirements of truth sometimes demand that these should neither be unnoticed nor uncondemned.

